# Focus on the Primary Prevention of Intrauterine Adhesions: Current Concept and Vision

**DOI:** 10.3390/ijms22105175

**Published:** 2021-05-13

**Authors:** Wen-Ling Lee, Chia-Hao Liu, Min Cheng, Wen-Hsun Chang, Wei-Min Liu, Peng-Hui Wang

**Affiliations:** 1 Department of Medicine, Cheng-Hsin General Hospital, Taipei 112, Taiwan; johnweiwang@gmail.com; 2 Department of Nursing, Oriental Institute of Technology, Taipei 220, Taiwan; 3 Institute of Clinical Medicine, National Yang Ming Chiao Tung University, Taipei 112, Taiwan; mikeliuu@gmail.com (C.-H.L.); alchemist791025@gmail.com (M.C.); whchang818@gmail.com (W.-H.C.); 4 Department of Obstetrics and Gynecology, Taipei Veterans General Hospital, Taipei 112, Taiwan; 5 Department of Nursing, Taipei Veterans General Hospital, Taipei 112, Taiwan; 6 Department of Obstetrics and Gynecology, Taipei Medical University Hospital, Taipei 110, Taiwan; weimin@tmu.edu.tw; 7 Female Cancer Foundation, Taipei 104, Taiwan; 8 Department of Medical Research, China Medical University Hospital, Taichung 404, Taiwan

**Keywords:** endometrium, hysteroscopic surgery, intrauterine adhesion, pathophysiology, prevention

## Abstract

Intrauterine adhesion (IUA), and its severe form Asherman syndrome (Asherman’s syndrome), is a mysterious disease, often accompanied with severe clinical problems contributing to a significant impairment of reproductive function, such as menstrual disturbance (amenorrhea), infertility or recurrent pregnancy loss. Among these, its correlated infertility may be one of the most challenging problems. Although there are many etiologies for the development of IUA, uterine instrumentation is the main cause of IUA. Additionally, more complicated intrauterine surgeries can be performed by advanced technology, further increasing the risk of IUA. Strategies attempting to minimize the risk and reducing its severity are urgently needed. The current review will expand the level of our knowledge required to face the troublesome disease of IUA. It is separated into six sections, addressing the introduction of the normal cyclic endometrial repairing process and its abruption causing the formation of IUA; the etiology and prevalence of IUA; the diagnosis of IUA; the classification of IUA; the pathophysiology of IUA; and the primary prevention of IUA, including (1) delicate surgical techniques, such as the use of surgical instruments, energy systems, and pre-hysteroscopic management, (2) barrier methods, such as gels, intrauterine devices, intrauterine balloons, as well as membrane structures containing hyaluronate–carboxymethylcellulose or polyethylene oxide–sodium carboxymethylcellulose as anti-adhesive barrier.

## 1. Introduction

The endometrium contains two main structural layers: an underlying stable basal layer (basal membrane), named the stratum basalis, and an upper dynamic and functional layer named the stratum functionalis [1]. The latter can be further separated into the thick stratum spongiosum and the thin stratum compactum [2]. The stratum functionalis is the portion shed during menstruation due to the withdrawal of estrogen and progesterone action in the absence of implantation and following pregnancy. This process is governed strictly and tightly by a complex cascade of endocrine along with paracrine signaling within the endometrium [3]. The basal layer, containing the stroma, basal portions of the glands, supporting vasculature, and various immune cell populations, such as natural killer (NK) cells, neutrophils, macrophages, and lymphocytes (T or B cells or mast cells) during menstruation, remains intact and provides a source of cells giving rise to a renewed stratum functionalis following menstruation [4]. The repair process of the endometrium is similar to the classical wound healing process, with some differences, including three separate, continuous and overlapping steps: the hemostasis/inflammatory phase, the proliferative phase, and the remodeling phase [5,6,7,8,9,10]. Naturally, the endometrium is tightly controlled by the orchestration of female sex hormones to complete its cyclic change, which is initiated in the proliferative phase and transforms into the secretory phase. All are for the preparation for embryo implantation, and this period of endometrial change is especially critical, often called uterine receptivity [11,12,13]. Endometrial tissue destruction (menstrual breakdown) and re-epithelialization (repair) occur simultaneously [1]. The endometrial re-epithelialization needs estrogen to stimulate glandular (the luminal epithelium) and stromal (the parenchymal endometrial stroma cells) regeneration [3]. Therefore, menstruation demonstrates that two opposing processes of tissue destruction and repair occur simultaneously in an inflammatory environment [3]. A purulent inflammatory reaction results in extensive destruction, which is not good for wound healing; however, under the estrogen’s function, the modulation of immune cell activity and tissue destruction and regeneration during menstruation will accelerate the repair process and facilitate the scar-free healing of the endometrium [3].

**Figure 1:**
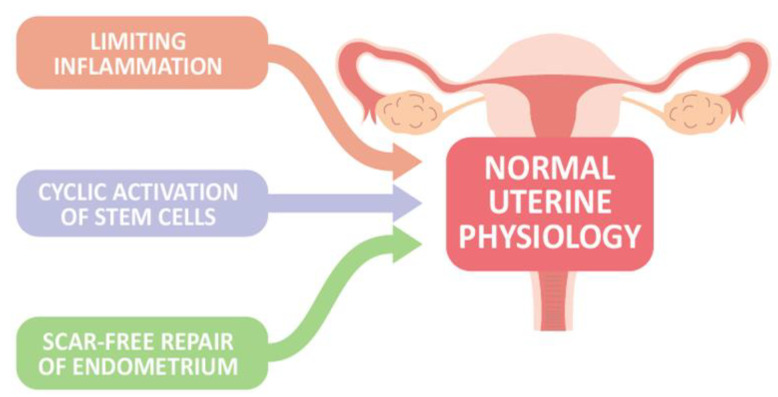
Three proposed key components of endometrial biology are present to maintain normal uterine physiology.

In the absence of conception and implantation, endometrial stroma cells sense hormone withdrawal, and upregulate intracellular signaling and the release of inflammatory and growth factors, contributing to vasoconstriction of the uterine vessels, recruitment of inflammatory and immune cells as well as relatively immune-privileged endometrial mesenchymal stem cells, and propagation of the menstrual cascade to aid in the regeneration and repairing process after menstruation, in concert with processes such as epithelial–mesenchymal transition/mesenchymal–epithelial transition (EMT/MET) and Wnt signaling, to restore endometrial homeostasis [14]. Furthermore, the activation of endometrial epithelial progenitor cells (N-cadherin+, E-cadherin+, and vimentin+ cells) and perivascular mesenchymal stem cells located in the basal layer near the endometrial–myometrial border, as well as within the functional layer, possibly involving Wnt or Notch signaling system, drives cellular replacement in the glands and stroma layer, respectively, to finish endometrial regeneration and maintain the endometrial integrity [3]. Taken together, at least three key components of endometrial biology are present for maintaining normal uterine physiology, including (1) limiting inflammation to prevent excessive tissue destruction, (2) cyclic activation of stem cells for endometrial regeneration, and (3) scar-free repair of the endometrium following menstrual shedding (Figure 1). However, when the healing process is interrupted, endometrial integrity is lost.

Endometrial integrity can be destroyed by many factors, such as surgery, intrauterine instrumentation, infection, and others. Cesarean section (C/S) is very common among women of childbearing age [15]. The endometrium after this type of injury is often recovered uneventfully, and rarely results in the development of sequelae. A possibly key reason is that the C/S procedure does not cause much trauma on the basal membrane. However, sometimes, the repairing of the endometrium after C/S is interrupted, resulting in C/S scar formation. The clinical symptoms of C/S scarring vary greatly, ranging from no symptoms to troublesome and life-threatening diseases. The symptoms include intermittent spotting or persistent bleeding, inability of implantation (subfertility, infertility, repeated miscarriage, or C/S scar ectopic pregnancy), or abnormality of implantation, such as placenta previa, placenta accrete, and placenta increta [16,17,18,19,20,21,22]. All of these might significantly increase the risk of pregnancy-related morbidity and mortality [19], including uterine rupture and postpartum hemorrhage (PPH). The latter can occur at any time of pregnancy or during labor.

More severe injury might occur in the uterine cavity, and with trauma reaching deep into the basal layer of the endometrium, the healing of the endometrium will be significantly impaired. In cases of extreme severity, the endometrial lining might be partially or totally absent, contributing to the approximation and fusion of surfaces of opposing uterine walls. As a result, IUA or Asherman syndrome (Asherman’s syndrome) develops [23,24,25,26,27,28,29,30,31,32].

## 2. Etiology and Prevalence of Intrauterine Adhesion (IUA)

Posttraumatic IUA was first reported by Dr. Heinrich Fritsch in 1894 [33]. Subsequently, Dr. Joseph Asherman published a series of papers to describe IUA in detail and depth [34]. There have been a large number of cases concerning IUA in the literature, with the result of more attention being paid to this syndrome [24]. Although Asherman syndrome is widely used to describe intrauterine adhesion with gravid uterus, Asherman syndrome may be better represented as a case of the total obliteration of the uterine cavity accompanied with amenorrhea. IUA might be a more appropriate term to describe all sequelae after intrauterine trauma, from minimal or marginal IUA to the complete obliteration of the uterine cavity and/or the inner walls of cervix [26,27].

The exact prevalence or real incidence of IUA is unknown, and may be underestimated, partly because of a lack of symptoms or the presence of vague symptoms, and partly because of neglect leading to the disease not being discovered even in those patients with clear symptoms [27,28,29,30,31,32,33,34,35,36,37,38,39,40,41,42]; as well as partly because of the uncertain predisposing and causal factors [30,35]. Additionally, IUA varies with geographic location, study population, and the availability of investigations for diagnosis [29,36]. Many review articles are available in the literature to describe the prevalence of IUA according to the various clinical presentations and surgeries [26,27,28,29,30]. The occurrence of IUA may be low as 1–2% in patients with secondary amenorrhea; 1–4% in patients after C/S; 3–4% in women undergoing postpartum dilation and curettage (D&C); 6–7% of patients receiving D&C for early spontaneous abortion; 6–7% in patients with infertility; 6–24% in women treated with hysteroscopic metroplasty; 10–20% in patients with spontaneous abortion occurring less than twice; 10–20% in patients undergoing D&C of elective abortion; 20–30% in patients with postpartum D&C occurring more than twice; 20–30% in patients requiring uterine instrumentation performed between the second and fourth postpartum weeks [27,30,37,38,39,40,41,42]. The development of IUA may occur in more than one-third of patients, if these patients have certain clinical presentations, such as missed abortion, late spontaneous abortion D&C, retained products of conception, recurrent pregnancy loss, and hysteroscopic myomectomy [27,30,37,38,39,40,41,42]. 

## 3. Diagnosis of Intrauterine Adhesion (IUA)

The diagnosis of IUA can be made based on careful evaluation and clinical presentation, which includes one or more clinical features, such as amenorrhea, hypomenorrhea, subfertility, and recurrent pregnancy loss, and it may occur with abnormal placentation, and/or other pregnancy complications, including intrauterine growth restriction secondary to IUA [30]. Although the clinical feature of IUA vary greatly, there is no doubt that IUA is still largely idiosyncratic in many women [27]. IUA can be suggested by the aforementioned clinical symptoms or signs, and evaluated using imaging modalities of hysterosalpingography (HSG) (with specificity and sensitivity ranging from 25.6 to 98.1% and from 21.6 to 98.0%, respectively); hysteroscopy (the standard diagnostic tool); transvaginal ultrasound (TVS) with specificity and sensitivity ranging from 78.6 to 100% and from 91 to 100%, respectively; three-dimensional (3-D) or four-dimensional (4-D) ultrasound combined with or without SHG; saline-infusion sonohysterography (SHG) with specificity and sensitivity ranging from 97.3 to 98.9% and from 12.8 to 62.5%, respectively; contrast SHG with specificity and sensitivity of more than 90%; or magnetic resonance imaging (MRI) [26,27,28,29,30]. Of course, IUA can be confirmed via typical histopathological findings [26,27,28,29].

Among the tools used to diagnose IUA or to evaluate the severity of IUA, hysteroscopy [43], similar to the role of laparoscopy in the assistance of clinically uncertain diagnoses of pelvic abnormalities [44,45,46], is a gold standard, which is also frequently used in studies to compare other diagnostic tools [28,29]. Although hysteroscopy is widely used in the diagnosis of IUA, there are some limitations to hysteroscopy. Hysteroscopy cannot evaluate the patency of tubes, and additionally, if the uterine cavity is totally obliterated, the hysteroscope cannot be inserted into the uterine cavity, and it thus fails to offer any informative data for IUA [27]. Unenhanced TVS is not particularly helpful in detecting IUA [28], although some reports have shown that endometrial thickness might play an assistant role [47]. HSG offers an opportunity to evaluate both uterine cavity and tubal patency, but a deficiency of detailed filling defect information and the presence of a high false positive rate impede its value for IUA diagnosis [48].

Enhanced dynamic SHG with the infusion of normal saline or gel into the uterine cavity enables the visualization of the uterine cavity and possible tubal patency, possessing a high negative predictive value and acceptable positive predictive value compared to hysteroscopy [49]. Similarly to the patients with total obliterated uterine cavity, the aforementioned two tools (HSG, contrast SHG) also fail to provide additional information inside the uterine cavity [28,50]. Virtual hysteroscopy, or a 3-D and advanced 4-D virtual reconstruction of the uterine cavity, were able to evaluate IUA in the obliterated cavity [28]. MRI may be another alternative despite its high cost, limited availability, and unknown sensitivity and specificity in the diagnosis of IUA [27]. One report from Korea showed that 3-D ultrasound might be useful in the diagnosis of IUA because of its high correlation with hysteroscopic findings (morphological abnormalities, marginal irregularity, thinning defects, obliteration, fibrosis, and calcification) [51].

## 4. Classification of Intrauterine Adhesion (IUA)

The classification of IUA is important, since it communicates information, such as the extent of the adhesion and the most appropriate therapeutic regimen, and it offers a better prediction of the results after treatment [26,27,28,29,30]. Several classification systems of IUA have been proposed for the description of its severity [52,53,54]. The American Fertility Society (AFS) and the European Society of Gynecological Endoscopy (ESGE) provide classifications for IUA. The classification of AFS for IUA (1988) used three items, including the extent of the cavity involved, the type of adhesions, and the menstrual pattern (Table 1), to produce the following prognostic classification: stage I (mild), II (moderate) and III (severe) [53]. The ESGE IUA classification includes grade I (thin or filmy adhesion, easily ruptured by hysteroscopic sheath alone and normal cornual area), II (singular dense adhesion that cannot be ruptured by the hysteroscopic sheath, but a uterine cavity connected to a separate space, and visualization of both tubal ostia), IIa (occluding adhesion only in the region of the internal cervical os with normal upper uterine cavity), III (multiple dense adhesions connected to a separate space of the uterine cavity and unilateral obliteration of tubal ostia), IV (extensive dense adhesion with partial occlusion of the uterine cavity and tubal ostia), Va (extensive endometrial scarring and fibrosis in combination with grade I or II adhesions, accompanied by amenorrhea or pronounced hypomenorrhea), and Vb (extensive endometrial scarring and fibrosis in combination with grade III or IV adhesion, accompanied with amenorrhea), based on hysteroscopic and hysterographic findings [27,28,29,30,54], and this classification was modified in 2017 [55].

Besides the aforementioned classification of IUA based on AFS and ESGE, there are at least six additional classification systems available for clinical practice [55], and these have been reported by the following authors: March et al. [27,56]; Hamou et al. [57]; Valle and Sciarra [58]; Donnez and Nisolle [59]; Nasr et al. [52]. However, to date, no data are available from any comparative analyses of these classification systems as regards which one is better [28]. One issue over which there is no argument is that clinical history had better be included into the classification system, given its good correlation with prognosis [29].

## 5. Pathophysiology of Intrauterine Adhesion

The basic histological finding of IUA is endometrial fibrosis. The stroma is largely replaced by avascular fibrous tissue and spindle-shaped myofibroblasts. The endometrial glands are replaced by inactive cubo-columnar endometrial epithelium, which cannot distinguish stratum functionalis from stratum basalis [28,29]. Additionally, this inactive single layer of cubo-columnar epithelium barely responds to hormonal stimulation. Finally, fibrotic synechiae form across the entire uterine cavity [60,61,62,63]. According to Dr. Foix’s classification [60], three types of IUA have been proposed, including (1) the most common type involves avascular fibrous strands joining the uterine wall, although sometimes, thin-walled telangiectatic vessels can be found in this avascular fibrous strand and calcification or ossification can be found in the stroma area, accompanied with spare and inactive or cystically dilated glands; (2) muscular adhesion composed of collagen bundles, fibrous strips, or muscle with the same characteristics as normal myometrium, of which the percentage of fibrous tissue is more than 50–80% in the biopsy specimens; and (3) a sclerotic, atrophic endometrium [60]. Han and Du summarized the pathological changes taking place in IUA, including endometrial fibrosis, endometrial scarring, the loss or thinning of the endometrium with different degrees of damage to the basal layer, atrophic glands, a lack of vascular stromal tissue and hypoxia, and a pale microenvironment in the adhesion area [62].

**Figure 2:**
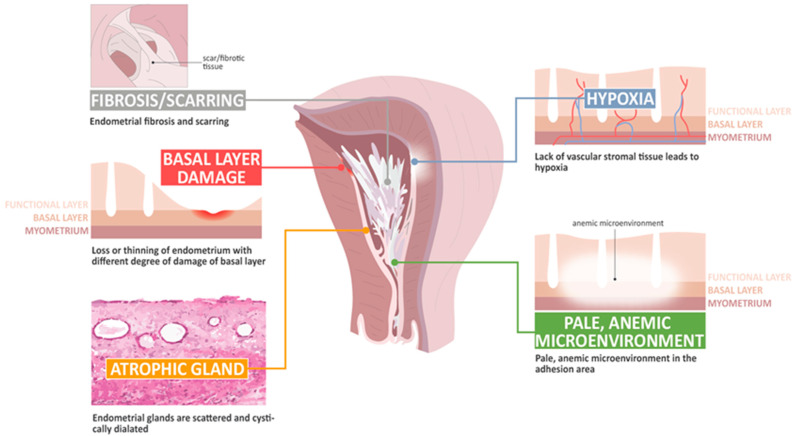
The pathophysiology of change in intrauterine adhesion.

IUA presents a challenge to the endometrial model of scar-free wound healing, as it would appear that several postulated mechanisms that allow for scar-free regeneration and repair are lost, including hypoxic injury, unbalanced inflammatory processes, decreased angiogenesis, the disturbance of immune and molecular mechanisms, an unregulated EMT, aberrant myofibroblast differentiation, bizarre stem cell regeneration, and interrupted normal endometrial cell proliferation [63,64,65,66,67,68,69,70,71,72]. However, evidence has shown the presence of distinct genetic profiles between patients with and without IUA, suggesting that IUA might not just be a representative of an iatrogenic disease, but also the result of a genetic predisposition [72]. Thus, a greater understanding of the pathophysiology of IUA might better elucidate the repairing of the defect behind IUA, and perhaps lead to the establishment of prevention and treatment strategies. Figure 2 shows several mechanisms postulated to be involved in the development of IUA.

## 6. Primary Prevention of Intrauterine Adhesion (IUA)

When IUA develops, the status is more complicated, because it is hard to predict the therapeutic outcome after the adhesiolysis of IUA. The treatment of IUA often needs surgical intervention, and most involve a hysteroscopic approach. However, adhesiolysis by hysteroscopy is often accompanied by higher complication and recurrence rates. All this suggests the paramount importance of primary prevention of IUA. Therefore, efforts should be made to minimize the risk of occurrence of IUA. IUA seems to be a frequent and major long-term complication in patients who undergo hysteroscopic surgery for certain diseases (submucosal myomas, adhesiolysis, septum or fibrosis of the intrauterine cavity [2,46,47,48,49], or repeated abortion surgery [27]. The risk of IUA after hysteroscopic myomectomy was determined to be as much as 31 to 78% [2,46,47,48,49], and over 30% after repeated abortion surgery, for which the times of the abortion surgery were strongly correlated with the risk of IUA development [27].

**Figure 3:**
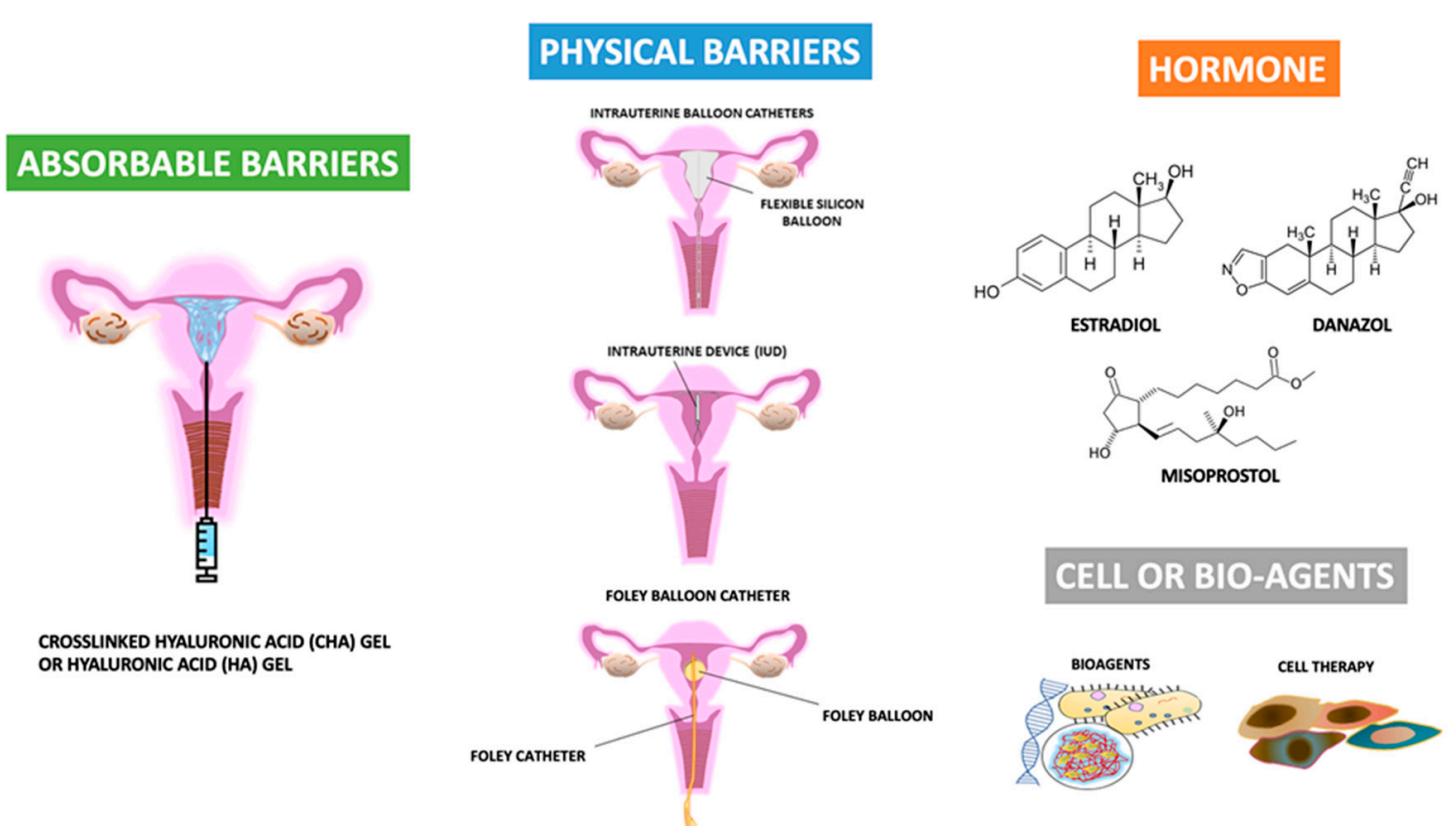
The recent development of agents available for the primary prevention of intrauterine adhesions after uterine surgery.

Many strategies or principles for decreasing the risk of developing IUA, such as the reduced use of electrosurgery and the minimization of trauma to the healthy endometrium and myometrium, have been proposed [73,74,75,76,77,78,79]. Additionally, many biomaterial agents, barrier methods, and cell or bio-agent therapies have been applied [14,16,24,27,28,29,30,31,35,37,40,43,44,62,63,64,65,66,67,68,69,70,71,72,73]. Figure 3 shows the recent development of agents available for the primary prevention of IUA after uterine surgery.

### 6.1. Surgical Techniques

First, the impact of surgical technology on the development of IUA should be considered, although it is unknown whether one technique is better than another [2], partly because of the presence of many confounding uncontrolled variables, meaning the data were derived with difficulty [73]. The most frequently discussed areas of surgical technology fit into five categories: (1) types of intrauterine instrumentation, (2) types of energy (bipolar or unipolar systems), (3) types of distending media (normal saline, dextran water or glycine), (4) types of intrauterine pressure (low pressure versus high pressure), and (5) preoperative additional therapy or postoperative additional therapy. The first four categories are related to the surgical procedures directly. The last one is similar to the concept of adjuvant therapy, which is discussed later.

#### 6.1.1. Surgical Instrumentation

In terms of the choice of intrauterine instrumentation, the development of IUA is highest in women displaying submucosal myoma after hysteroscopic surgery. For example, submucosal myoma could be removed by a variety of surgical techniques. The most common tools in the clinical practice include hysteroscopic resectoscopy and hysteroscopic mechanical morcellation (known as hysteroscopic tissue removal) [79]. The latter incorporates two main systems: the Truclear (Medtronic, Minneapolis, MN, USA), and the MyoSure (Hologic, Marlborough, MA, USA), both of which have been approved by the Food and Drug Administration (FDA) [73,74,79]. In theory, hysteroscopic tissue removal might cause less damage to the endometrial cavity than resectoscopic myomectomy dose, although no comparison studies have been conducted to evaluate the incidence of IUA in women with submucosal myomas treated either by hysteroscopic resectoscopy or by hysteroscopic mechanical morcellation [73,74,75,76,77,78,79,80,81,82,83]. By contrast, a comparison study has been carried out on the management of women with placental remnants [80,84,85]. The results showed that there were no statistically significant differences in adverse events, tissue availability, short-term effectiveness or postoperative IUAs when hysteroscopic morcellation and bipolar loop resection for the removal of placental remnants were compared [79,82,83].

#### 6.1.2. Energy System during Hysteroscopic Surgery

In terms of energy systems, some reports favor the use of a bipolar system in place of a unipolar system during hysteroscopic surgery in order to decrease the risk of IUA formation [2]. Reports showed that the occurrence of IUA was 7.5% after hysteroscopic tissue removal using a bipolar energy system compared to 35% in those using a unipolar energy system [79], making bipolar systems the most widely used energy system during hysteroscopic surgery [80]. Additionally, the advantages of bipolar systems are further augmented by the lower risk of metabolic complications and fluid overload during operation [73,76]. We also adopted this type (a bipolar system) of device in our institution with an acceptable result [86]. However, recently, the use of a different application, such as a “cold loop” system, which is mediated by a unipolar energy system, in the management of women with submucosal myoma was reported to involve a relatively low risk of IUA formation (4.2%) [81]. One small series of eight women with menorrhagia, anemia, and infertility secondary to types 0, I and II submucosal myomas were treated with a hysteroscopy endo operative system (combination of cold graspers and electric loop), yielding a favorable reproductive outcome [87]. The authors believed that the cold surgery and fenestration method could minimize electrical and thermal damage to the endometrium surrounding the myoma, consequently reducing surgical risks [87]. That is to say that any electric energy device (unipolar/bipolar) may cause possible thermal damage, contributing to the development of an electric energy-free instrument, as shown in the aforementioned Section 6.1.1., addressing the surgical instrumentation. A recent systematic review tried to summarize the available evidence on the role of hysteroscopic tissue removal systems in the management of submucosal myomas [88]. The authors found that despite the introduction of hysteroscopic tissue removal systems into clinical practice, published data about their use in the management of women with submucosal myomas are still extremely limited [88]. Although the real cause is uncertain, it is possible that the surgeons do not favor this procedure due to its higher cost, considering that the complete treatment of type II submucosal myomas often requires both hysteroscopic tissue removal systems and classical resectoscoping in the operating theatre [88]. Taken together, we believe the best way to limit electronic device-related damage is to avoid excessive energy application to the endometrium, either directly or indirectly. Better visibility, precisely cutting, and limited operation time may further decrease the thermal effect secondary to the electronic device.

#### 6.1.3. Pre-Hysteroscopic Management

In terms of preoperative procedures carried out before hysteroscopic tissue removal, some may increase the risk of IUA development. The most common clinical situations include a relatively large submucosal myoma, and the management of retained placenta (placenta remnants) and/or abnormal placentation, such as placenta previa, accrete, increta, and percreta [21,36,78,79,80,81,82,83,84,85]. Preoperative uterine artery embolization (UAE) or a similar procedure performed by surgeons (uterine vessel occlusion (UVO) either by laparoscopy or by exploratory laparotomy) may be an example [89,90,91,92,93,94]. With the assistance of preoperative UAE or UVO, the procedure-related difficulty might be decreased, including less blood loss, easy resection, or morcellation; however, the incidence of IUA (18–30%) seemed to be similar to or higher than that in those treated via traditional hysteroscopic myomectomy without the aforementioned procedure [2]. The value of using UAE prior to hysteroscopic myomectomy is questionable, partly because of the possibility of an increased risk of IUA formation, and most importantly, UAE might be associated with a greater therapeutic challenge in further surgery for IUA (difficulty of intrauterine adhesiolysis) [2,92]. One study found that the outcome after hysteroscopic adhesiolysis was statistically significantly worse in women with UAE-related IUA than in those with IUA caused by surgical trauma [92]. The reason is unclear, but uterine ischemia (hypoxic injury, inflammation, and decreased angiogenesis) might be a direct cause.

By contrast, other strategies, such as medication (gonadotropin-releasing hormone agonist (GnRH agonist), selective progesterone receptor modulator (SPRM), and others) are frequently used in the preoperative preparation before hysteroscopic tissue removal, including hysteroscopic myomectomy, because of the advantages of increased hemoglobin level and the shrinkage of tumor size, but the majority of articles are limited to discussing the feasibility and safety of these strategies in the assistance of complete hysteroscopic tissue removal, such as the percentage of myomas resected, the duration of surgery, the fluid deficit, and complications [95,96,97,98,99]. There is still a lack of data on the risk of IUA when the patients were treated with medication before hysteroscopic myomectomy, but some reports have shown no significantly gross change in the endometrium during hysteroscopy between the no pretreatment and pretreatment groups [42,100]. Taskin and colleagues found that IUA is the major long-term complication of operative hysteroscopy, with frequency and severity dependent on the pathology initially treated, and pretreatment-induced hypoestrogenism did not affect the frequency and severity of IUA formation [42]. Another report showed a similar rate of postoperative adhesion formation after abdominal myomectomy in patients undergoing GnRH agonist pretreatment compared to in patients without pretreatment [100]. To date, no evidence suggests a correlation between pretreatment and the development of IUA in patients after hysteroscopic tissue removal, hysteroscopic myomectomy, or repeated abortion surgery.

The recognition of the importance of IUA development after hysteroscopic surgery or intrauterine surgery lead to the need for preventing its occurrence. In addition, subsequent surgery (intrauterine adhesiolysis) in women with adhesions is more difficult, often takes longer, and is associated with a higher risk of complication and recurrence rate [56,101,102]. Therefore, besides the role of surgical technology in IUA as shown above, several measures have been proposed in an effort to decrease IUA after hysteroscopic surgery or repeated intrauterine surgery, such as abortion surgery, based on the four strategies. One involves the avoidance of over- or purulent inflammatory processes or over-traumatic injury; the second is a barrier method to prevent the attachment of the bilateral traumatic surface; the third is the enhancement of regeneration ability; the last is early intervention via repeat hysteroscopy to remove the thin or immature adhesion band (Figure 3). The tools include agents (drugs) or physical (mechanical) barriers to cover the uterine lining side, or separate the opposing sides of the uterine linings immediately after hysteroscopic surgery; the prescription of estrogen use postoperatively, postoperative gonadotropin-releasing hormone agonist injection, or immediate postoperative antibiotics use; and the use of biomaterials or new technologies such as cellular therapy [5,6,7,8,9,10,14,23,24,27,28,29,30,31,32,35,36,37,39,40,41,42,43,56,57,58,59,62,63,64,65,66,67,68,69,70,71,86,101,102,103,104,105,106,107,108,109,110,111,112,113,114,115,116,117,118,119,120,121,122,123,124,125,126,127,128,129,130,131,132,133,134,135,136,137,138,139,140,141,142,143,144,145,146,147,148,149,150,151,152,153,154,155,156]. However, the majority of new technologies seem to be a long way from routine clinical use. Additionally, some useful strategies based on the prevention of recurrent IUA or the repair of the defective uterine wall have not been included in the current review. Moreover, the combination of two or more methods in the prevention of IUA formation after primary uterine surgery was also excluded.

### 6.2. Barrier Methods

Barrier is one of the frequently used tools in the prevention of adhesion, since in theory, the separation of two opposite sides with a rough surface can prevent the contact of both sides, and subsequently decrease the risk of adhesion between the two. A barrier can be achieved by two strategies; one uses agents and the other is physical or mechanical. Agents act as a barrier, can be solid form, liquid form (hydroflotation agents) or gel form, and the components include polyethylene oxide–sodium carboxymethylcellulose gel and crosslinked hyaluronic acid (CHA) gels [71,81,82,96,97,98,99,104,105,106,107,108,109,110,111,112,113,114,115,116,117,118,120,148,149,154,155,156]. Physical or mechanical barriers include intrauterine suitable balloon catheters, Foley balloon catheters, Malecot catheters, silicone sheets, and intrauterine devices (IUD) [73,103,104,105,106,107,108].

#### 6.2.1. Gels as Anti-Adhesive Agents

Gel might be one of most convenient agents for application in a limited and irregular space, such as the intrauterine space, and most gel agents commonly contain derivatives of hyaluronic acid (HA) with other main components, such as sodium D-glucuronate and N-acetyl-glucosamine, which is a linear polysaccharide with 25,000 repeating disaccharide units, composed of major supportive and protective components in a vitreous body, saliva, synovial fluid, cartilage, skin, and umbilical cord [157,158,159,160,161,162,163]. Earlier reports have shown that the application of CHA gel has reduced the severity of postoperative IUA after hysteroscopic procedures [86,109,111,112]. A recent prospective randomized controlled trial was conducted to evaluate whether the intrauterine application of HA gel after D&C may improve reproductive outcomes, and the results showed that patients in the HA treatment group had not only shortened times to conception leading to a live birth (21.9 vs. 36.2 months), lower risks of reduced menstrual blood loss (7.5% vs. 20.3%), and lower risks of dysmenorrhea (14.9% vs. 34.4%), but also a higher ongoing pregnancy rate (74.6% vs. 67.2%), than patients without HA treatment do, suggesting that the application of HA gel following D&C performed after a miscarriage is beneficial for subsequent reproductive performance in women with at least one previous D&C [116].

There are many systematic reviews and meta-analyses available that evaluate the effectiveness of the using HA in the primary prevention of IUA after intrauterine surgery or miscarriage, and the effects not only demonstrate lower incidence, but also show a reduced severity, of IUA in HA-treated patients.

Zheng and colleagues attempted to systematically evaluate the efficacy of hyaluronic acid gel in preventing IUA following intrauterine operation [125]. Seven randomized controlled clinical studies have been performed [96,104,109,114,124,125,126], enrolling 952 patients after intrauterine operation. The results showed that the use of hyaluronic acid gel significantly reduced the incidence of IUA with a relative risk (RR) of 0.42 (95% confidence interval (CI) 0.30–0.57, *p* < 0.001) [125]. In addition, the score for IUA after intrauterine operation was also statistically significantly decreased in patients with the use of hyaluronic acid gel, with a mean difference (MD) −1.29, 95% CI from −1.73 to −0.84, and *p* < 0.001 [125]. The authors also found that the protective role of hyaluronic acid against the development of IUA was not affected either by diseases or by types of intrauterine surgery. In terms of abortion, the RR was 0.48 (95% CI 0.29–0.78, *p* = 0.003). In terms of IUA, the RR was 0.38 (95% CI 0.21–0.67, *p* < 0.001). Similar to the current study, in terms of submucosal myoma, endometrial polyps or mediastinum uterus, the RR was also statistically significant decreased (RR 0.40, 95% CI 0.18–0.90, *p* = 0.03). Considering the type of intrauterine surgery, the effectiveness of hyaluronic acid gel in the reduction of IUA formation was also statistically apparent, with an RR of 0.40 (95% CI 0.26–0.62, *p* < 0.001) in the abortion surgery and 0.44 (95% CI 0.28–0.68, *p* < 0.001) in the hysteroscopic surgery [125]. The most promising result of this meta-analysis showed that hyaluronic acid gel was beneficial for future pregnancy rates after intrauterine surgery (RR 1.94, 95% CI 1.46–2.60, *p* < 0.001) [125].

Another earlier meta-analysis was conducted to evaluate the effectiveness of the use of HA gel to prevent IUA after miscarriage [123]. The results of a total of four studies [109,114,128,129] enrolling 625 patients showed that HA gel significantly reduced the incidence of IUA after miscarriage with a relative risk (RR) of 0.44 and a 95% confidence interval (95% CI) ranging from 0.29 to 0.67 (*p* = 0.0001), and it also significantly reduced the severity of IUA after miscarriage, with a standardized mean difference (SMD) of −0.68 and a 95% CI ranging from −1.08 to −0.28 in the IUA scores (*p* = 0.0008) [123]. In agreement with our aforementioned findings, this meta-analysis also found that HA gel significantly reduced the incidence of moderate and severe IUA after miscarriage (RR 0.18, 95% CI 0.07–0.47, *p* = 0.0004), but seemed to have no effect on the reduced incidence of mild-form IUA (RR 0.77, 95% CI 0.42–1.19, *p* = 0.19) [123]. Yan and Xu also found that HA corresponded to a relatively high preventive effect against severe IUAs, as using HA was superior to no treatment (logOR (odds ratio) −2.44, 95% CI from −3.95 to −0.92), and furthermore, the IUA scores in the HA treatment group showed an SMD of −1.25 (95% CI from −1.52 to −0.97) compared to those in the no-treatment group [24].

There are several types of HA available in clinical practice. Some studies were conducted to compare the anti-adhesive effects between the different modifications of HA [89,146]. Although the data showed the effectiveness of different modifications of HA in the prevention of IUA formation, our previous study suggested a high concentration of HA may provide better therapeutic effects, given the lower severity of IUA seen in the high-concentration HA group [86].

#### 6.2.2. Intrauterine Device (IUD) or Intrauterine Balloon as Anti-Adhesive Barrier

In theory, the application of an IUD is effective in the prevention of IUA formation after intrauterine surgery. However, the placement of an IUD is frequently applied for the prevention of subsequent adhesion formation after adhesiolysis, and this procedure is considered as the standard method to maintain the integrated uterine contour (cavity) [105]. Therefore, compared to the use of HA for the primary prevention of IUA, studies focusing on the use of IUD are relatively few in number [24]. Tonguc and colleagues in 2010 found that the rates of IUA were 10.5%, 0%, 12% and 5.3%, respectively, in the IUD placement, estrogen alone, combination of IUD and estrogen, and no treatment groups, suggesting that neither IUD placement nor estrogen treatment, nor both together, were found to prevent intrauterine adhesions or facilitate pregnancy in patients after hysteroscopic uterine septum resection [145].

Yu and colleagues enrolled 238 patients who underwent hysteroscopic uterine septum resection to investigate the clinical efficacy of postoperative estrogen therapy, IUD, and intrauterine balloon use in preventing IUA, and the results showed that the IUA rates were 22%, 28.8%, 26.7%, and 24.1%, respectively, in the estrogen, IUD, intrauterine balloon, and no treatment groups at one month postoperatively [146]. At the end of 3 months, the IUA rates among the four groups were 0%, 1.7%, 1.3%, and 3.4%, respectively [146]. Therefore, the authors commented that the uses of postoperative estrogen therapy, IUD or intrauterine balloon were not significantly different in reducing the incidence of postoperative IUA formation [146]. Nevertheless, given the retrospective nature of this cohort study, the conclusion should be considered preliminary, and a further prospectively planned randomized study is needed to confirm the findings. In fact, as early as 1989, Vercellini’s group had shown that IUD insertion and hormone therapy after hysteroscopic metroplasty were not needed for to prevent IUA formation in women who underwent hysteroscopic metroplasty [147].

To summarize all the above-mentioned findings, the primary prevention of IUA using an IUD or intrauterine balloon with or without estrogen seemed to be not effective in the prevention of IUA formation in women who underwent hysteroscopic uterine septum resection.

#### 6.2.3. Membrane Structure Containing Hyaluronate–Carboxymethylcellulose (ACH or CH) or Polyethylene Oxide–Sodium Carboxymethylcellulose (Intercoat) as the Anti-Adhesive Barrier

A meta-analysis conducted by Yan et al. found that an alginate hyaluronate–carboxymethylcellulose (ACH) membrane was the most effective adjuvant treatment in preventing IUA incidence [24]. In addition, the no-treatment control was far inferior to Intercoat (logOR 1.58, 95% CI 0.25–2.90) [24]. According to the review by Yan and Xu, the most effectively adjuvant therapy in the primary prevention of IUAs may be ACH, since it was reported to be more than 93.3% effective [24].

## 7. Future Vision of IUA Management

Primary prevention is the most important issue for women who need uterine surgery, especially for those women of reproductive age suffering from intrauterine lesions. Even though much evidence suggests that the risk of developing IUA can be reduced using the aforementioned strategies, it is well known that IUA cannot be totally avoided. Therefore, subsequent IUA management is considered necessary when IUA occurs. Thus far, there have been many publications addressing the topic of the management of IUA [23,24,26,27,28,29,30,35,40,43,57,58,59,62,64,131,132,133,134,135,136,138,139,140,141,142,155,164,165,166,167,168].

As shown in Figure 3, bioagents, cellular therapy, and three-dimensional biotechnology may be useful in the management of women with IUA. Re-starting endometrial regeneration and scar-free repair of the uterine cavity is the key factor for the restoration of normal reproductive function in women with IUA. Recently, the regenerative potential of stem cells has demonstrated improved outcomes in terms of fertility and fibrosis in both mice and humans with IUA [28,164,165,166]. Stem cells impact tissue repair by honing in on the injured site, recruiting other cells by secreting chemokines, modulating the immune system, differentiating into different types of cells, and differentiating into daughter cells [164]. We welcome more research addressing this issue to provide evidence concerning effectiveness and long-term safety.

## 8. Conclusions

IUA is still a challenging disease, although it is often neglected. The risk of the development of IUA is relatively high in women undergoing hysteroscopic myomectomy. Primary prevention might permit a more effective reduction in IUA formation, thus helping avoid the subsequent impairment of reproductive performance. To summarize all the findings, the primary prevention of IUA using an IUD or intrauterine balloon with or without estrogen did not attain statistical significance in preventing IUA formation in women who underwent hysteroscopic uterine septum resection. Our previous findings demonstrated that crosslinked hyaluronic acid gels are highly recommended for the prevention of intrauterine adhesions [86]. A pooled analysis of two studies that limited the use of ACHA in 119 women showed that the application of ACHA gel for the primary prevention of IUA in patients after hysteroscopic myomectomy led to a statistically significant reduction in the development of IUA postoperatively (OR 0.285, 95% CI 0.116–0.701, *p* = 0.006) [148]. All this suggests that using ACHA gel in patients after hysteroscopic myomectomy could significantly reduce de novo IUA. Therefore, any strategy, such as the use of anti-adhesive agents, including HA-related biomaterials like CHAP and auto-cross-linked hyaluronic acid (ACP) gels, or other barrier methods, such as using membranes or Intercoat containing alginate hyaluronate–carboxymethylcellulose, hyaluronate–carboxymethylcellulose, or polyethylene oxide–sodium carboxymethylcellulose, should be considered, although more studies are needed to provide supporting evidence.

## Figures and Tables

**Table 1:** Classification of the American Fertility Society scoring system for intrauterine adhesion.

Scoring/Characteristics	Extent Cavity Involved	Type of Adhesions	Menstrual Pattern
1	<1/3	Filmy	
2	1/3~2/3	Filmy~Dense	Hypomenorrhea
4	>2/3	Dense	Amenorrhea

Mild (stage I): scoring from 1 to 4; moderate (stage II): scoring from 5 to 8; severe (stage III): scoring ≥ 9.

## Abbreviations

The following abbreviations are used in this manuscript:

IUA	intrauterine adhesion or intrauterine adhesions
EMT	epithelial–mesenchymal transition
MET	mesenchymal–epithelial transition
C/S	cesarean section
HSG	hysterosalpingography
TVS	transvaginal ultrasound
3-D	three-dimensional
4-D	four-dimensional
SHG	sonohysterography
MRI	magnetic resonance imaging
D&C	dilation and curettage
AFS	the American Fertility Society
ESGE	the European Society of Gynecological Endoscopy
HTR	hysteroscopic tissue removal
FDA	the Food and Drug Administration
UAE	uterine artery embolization
GnRH	gonadotropin-releasing hormone
SPRM	selective progesterone receptor modulation
CHA	crosslinked hyaluronic acid
IUD	intrauterine devise
CHAP	crosslinked hyaluronic acid plateform
HA	hyaluronic acid
RR	relative risk
95% CI	95% confidence interval
SMD	standardized mean difference
OR	odd ratio

## References

[1] Owusu-Akyaw A., Krishnamoorthy K., Goldsmith L.T., Morelli S.S. (2019). The role of mesenchymal-epithelial transition in endometrial function. Hum. Reprod. Update.

[2] Torres-De La Roche L.A., Campo R., Devassy R., Di Spiezio Sardo A., Hooker A., Koninckx P., Urman B., Wallwiener M., De Wilde R.L. (2019). Adhesions and anti-adhesion systems highlights. Facts Views Vis. ObGyn.

[3] Evans J., Salamonsen L.A., Winship A., Menkhorst E., Nie G., Gargett C.E., Dimitriadis E. (2016). Fertile ground: Human endometrial programming and lessons in health and disease. Nat. Rev. Endocrinol..

[4] Weimar C.H., Macklon N.S., Post Uiterweer E.D., Brosens J.J., Gellersen B. (2013). The motile and invasive capacity of human endometrial stromal cells: Implications for normal and impaired reproductive function. Hum. Reprod. Update.

[5] Jiang D., Rinkevich Y. (2020). Scars or regeneration?—Dermal fibroblasts as drivers of diverse skin wound responses. Int. J. Mol. Sci..

[6] El Ayadi A., Jay J.W., Prasai A. (2020). Current approaches targeting the wound healing phases to attenuate fibrosis and scarring. Int. J. Mol. Sci..

[7] Akita S. (2019). Wound repair and regeneration: Mechanisms, signaling. Int. J. Mol. Sci..

[8] Ogawa R. (2018). Recent advances in scar biology. Int. J. Mol. Sci..

[9] Wang P.H., Huang B.S., Horng H.C., Yeh C.C., Chen Y.J. (2018). Wound healing. J. Chin. Med. Assoc..

[10] Horng H.C., Chang W.H., Yeh C.C., Huang B.S., Chang C.P., Chen Y.J., Tsui K.H., Wang P.H. (2017). Estrogen effects on wound healing. Int. J. Mol. Sci..

[11] Marquardt R.M., Kim T.H., Shin J.H., Jeong J.W. (2019). Progesterone and estrogen signaling in the endometrium: What goes wrong in endometriosis?. Int. J. Mol. Sci..

[12] Wang P.H. (2018). Endometrial receptivity and adenomyosis. Taiwan. J. Obstet. Gynecol..

[13] Lai T.H., Chang F.W., Lin J.J., Ling Q.D. (2018). Endometrial L-selectin ligand is downregulated in the mid-secretory phase during the menstrual cycle in women with adenomyosis. Taiwan. J. Obstet. Gynecol..

[14] Chen J.X., Yi X.J., Gu P.L., Gao S.X. (2019). The role of KDR in intrauterine adhesions may involve the TGF-β1/Smads signaling pathway. Braz. J. Med. Biol Res..

[15] Kwack J.Y., Lee S.J., Kwon Y.S. (2021). Pregnancy and delivery outcomes in the women who have received adenomyomectomy: Performed by a single surgeon by a uniform surgical technique. Taiwan. J. Obstet. Gynecol..

[16] Nakamura K., Kusama K., Suda Y., Fujiwara H., Hori M., Imakawa K. (2020). Emerging role of extracellular vesicles in embryo-mternal communication throughout implantation processes. Int. J. Mol. Sci..

[17] Fujiwara H., Ono M., Sato Y., Imakawa K., Iizuka T., Kagami K., Fujiwara T., Horie A., Tani H., Hattori A. (2020). Promoting Roles of embryonic signals in embryo implantation and placentation in cooperation with endocrine and immune systems. Int. J. Mol. Sci..

[18] Wu Y., Sun L.F., Si Y.N., Luan X.L., Gao Y.M. (2021). Clinical efficacy analysis of different therapeutic methods in patients with cesarean scar pregnancy. Taiwan. J. Obstet. Gynecol..

[19] Yildirim G.Y., Koroglu N., Akca A., Talmac M., Dikmen S., Yıldırım G., Polat I., Ozdemir I. (2021). What is new in peripartum hysterectomy? A seventeen year experience in a tertiary hospital. Taiwan. J. Obstet. Gynecol..

[20] Zhang L.P., Wang M., Shang X., Zhang Q., Yang B.J., Xu Y., Li J.H., Feng L.M. (2020). The incidence of placenta related disease after the hysteroscopic adhesiolysis in patients with intrauterine adhesions. Taiwan. J. Obstet. Gynecol..

[21] Horng H.C., Lai M.J., Chang W.H., Wang P.H. (2021). Placenta accreta spectrum (PAS) and peripartum hysterectomy. Taiwan. J. Obstet. Gynecol..

[22] Guarga Montori M., Álvarez Martínez A., Luna Álvarez C., Abadía Cuchí N., Mateo Alcalá P., Ruiz-Martínez S. (2021). Advanced maternal age and adverse pregnancy outcomes: A cohort study. Taiwan. J. Obstet. Gynecol..

[23] Li B., Zhang Q., Sun J., Lai D. (2019). Human amniotic epithelial cells improve fertility in an intrauterine adhesion mouse model. Stem. Cell Res. Ther..

[24] Yan Y., Xu D. (2018). The Effect of adjuvant treatment to prevent and treat intrauterine adhesions: A network meta-analysis of randomized controlled trials. J. Minim. Invasive Gynecol..

[25] Muneer A., Macrae B., Krishnamoorthy S., Zumla A. (2019). Urogenital tuberculosis—Epidemiology, pathogenesis and clinical features. Nat. Rev. Urol..

[26] Yu D., Wong Y.M., Cheong Y., Xia E., Li T.C. (2008). Asherman syndrome-one century later. Fertil. Steril..

[27] March C.M. (2011). Management of Asherman’s syndrome. Rerpod. Biomed. Online.

[28] Dreisler E., Kjer J.J. (2019). Asherman’s syndrome: Current perspectives on diagnosis and management. Int. J. Womens Health.

[29] Deans R., Abbott J. (2010). Review of intrauterine adhesions. J. Minim. Invasive Gynecol..

[30] Doroftei B., Dabuleanu A.M., Ilie O.D., Maftei R., Anton E., Simionescu G., Matei T., Armeanu T. (2020). Mini-review of the new therapeutic possibilities in Asherman syndrome—Where are we after one hundred and twenty-six years?. Diagnostics.

[31] Wang Y.Q., Song X.H., Wu S.L., Huang Y.Z., Yan L., Li C.Z. (2020). Comparison of autocross-linked hyaluronic acid gel and intrauterine device for preventing intrauterine adhesions in infertile patients: A randomized clinical trial. Gynecol. Minim. Invasive Ther..

[32] Van Wessel S., Hamerlynck T., Schutyser V., Tomassetti C., Wyns C., Nisolle M., Verguts J., Colman R., Weyers S., Bosteels J. (2021). Anti-adhesion gel versus no gel following operative hysteroscopy prior to subsequent fertility treatment or timed InterCourse (AGNOHSTIC), a randomised controlled trial: Protocol. Hum. Reprod. Open.

[33] Fritsch H. (1894). Ein Fall von volligen Schwund der Gebaumutterhohle nach Auskratzung. Zent. Gynaekol..

[34] Asherman J.G. (1948). Amenorrhoea traumatica (atretica). J. Obstet. Gynaecol. Br. Emp..

[35] Tu C.H., Yang X.L., Qin X.Y., Cai L.P., Zhang P. (2013). Management of intrauterine adhesions: A novel intrauterine device. Med. Hypothesis.

[36] Hooker A.B., Lemmers M., Thurkow A.L., Heymans M.W., Opmeer B.C., Brolmann H.A.M., Mol B.W., Huirne J.A.F. (2014). Systematic review and meta-analysis of intrauterine adhesions after miscarriage: Prevalence, risk factors and long-term reproductive outcome. Hum. Reprod. Update.

[37] Chiu C.S., Hwu Y.M., Lee R.K., Lin M.H. (2020). Intrauterine adhesion prevention with Malecot catheter after hysteroscopic myomectomy: A novel approach. Taiwan. J. Obstet. Gynecol..

[38] Liao W.L., Ying T.H., Shen H.P., Wu P.J. (2019). Combined treatment for big submucosal myoma with High Intensity Focused Ultrasound and hysteroscopic resection. Taiwan. J. Obstet. Gynecol..

[39] Yang J.H., Chen M.J., Chen C.D., Chen S.U., Ho H.N., Yang Y.S. (2013). Optimal waiting period for subsequent fertility treatment after various hysteroscopic surgeries. Fertil. Steril..

[40] Yang J.H., Chen M.J., Wu M.Y., Chao K.H., Ho H.N., Yang Y.S. (2008). Office hysteroscopic early lysis of intrauterine adhesion after transcervical resection of multiple apposing submucous myomas. Fertil. Steril..

[41] Mettler L., Schollmeyer T., Tinelli A., Malvasi A., Alkatout I. (2012). Complications of uterine fibroids and their management, surgical management of fibroids, laparoscopy and hysteroscopy versus hysterectomy, haemorrhage, adhesions, and complications. Obstet. Gynecol. Int..

[42] Taskin O., Sadik S., Onoglu A., Gokdeniz R., Erturan E., Burak F., Wheeler J.M. (2000). Role of endometrial suppression on the frequency of intrauterine adhesions after resectoscopic surgery. J. Am. Assoc. Gynecol. Laparosc..

[43] Di Guardo F., Della Corte L., Vilos G.A., Carugno J., Török P., Giampaolino P., Manchanda R., Vitale S.G. (2020). Evaluation and treatment of infertile women with Asherman syndrome: An updated review focusing on the role of hysteroscopy. Reprod. Biomed. Online.

[44] Passos I.M.P.E., Britto R.L. (2020). Diagnosis and treatment of Mullerian malformations. Taiwan. J. Obstet. Gynecol..

[45] Sao C.H., Chan-Tioplanco M., Chung K.C., Chen Y.J., Horng H.C., Lee W.L., Wang P.H. (2019). Pain after laparoscopic surgery: Focus on shoulder-tip pain after gynecological laparoscopic surgery. J. Chin. Med. Assoc..

[46] Lin S., Xie X., Guo Y., Zhang H., Liu C., Yi J., Su Y., Deng Q., Zhu W. (2020). Clinical characteristics and pregnancy outcomes of infertile patients with endometriosis and endometrial polyps: A retrospective cohort study. Taiwan. J. Obstet. Gynecol..

[47] Fedele L., Bianchi S., Dorta M., Vignali M. (1996). Intrauterine adhesions: Detection with transvaginal US. Radiology.

[48] Roma Dalfó A., Ubeda B., Ubeda A., Monzón M., Rotger R., Ramos R., Palacio A. (2004). Diagnostic value of hysterosalpingography in the detection of intrauterine abnormalities: A comparison with hysteroscopy. AJR Am. J. Roentgenol..

[49] Berridge D.L., Winter T.C. (2004). Saline infusion sonohysterography: Technique, indications, and imaging findings. J. Ultrasound. Med..

[50] Lee F.K., Lee W.L., Wang P.H. (2017). Is hysterosalpingography a good tool to confirm the patency of tubes. J. Chin. Med. Assoc..

[51] Kim M.J., Lee Y., Lee C., Chun S., Kim A., Kim H.Y., Lee J.Y. (2015). Accuracy of three dimensional ultrasound and treatment outcomes of intrauterine adhesion in infertile women. Taiwan. J. Obstet. Gynecol..

[52] Nasr A.L., Al-Inany H.G., Thabet S.M., Aboulghar M. (2000). A clinicohysteroscopic scoring system of intrauterine adhesions. Gynecol. Obstet. Investig..

[53] (1988). The American Fertility Society classifications of adnexal adhesions, distal tubal occlusion, tubal occlusion secondary to tubal ligation, tubal pregnancies, Müllerian anomalies and intrauterine adhesions. Fertil. Steril..

[54] Wamsteker K., Sutton C.D., Diamond M. (1998). Diagnostic hysteroscopy: Technique and documentation. Endoscopic Surgery for Gynecologists.

[55] AAGL Elevating Gynecologic Surgery (2017). AAGL Practice report: Practice guidelines on intrauterine adhesions developed in collaboration with the European Society of Gynaecological Endoscopy (ESGE). J. Minim. Invasive Gynecol..

[56] March C.M., Israel R., March A.D. (1978). Hysteroscopic management of intrauterine adhesions. Am. J. Obstet. Gynecol..

[57] Hamou J., Salat-Baroux J., Siegler A.M. (1983). Diagnosis and treatment of intrauterine adhesions by microhysteroscopy. Fertil. Steril..

[58] Valle R.F., Sciarra J.J. (1988). Intrauterine adhesions: Hysteroscopic diagnosis, classification, treatment, and reproductive outcome. Am. J. Obstet. Gynecol..

[59] Donnez J., Nisolle M., Donnez J. (1994). Hysteroscopic lysis of intrauterine adhesions (Asherman syndrome). Atlas of Laser Operative Laparoscopy and Hysteroscopy.

[60] Foix A., Bruno R.O., Davison T., Lema B. (1966). The pathology of postcurettage adhesions. Am. J. Obstet. Gynecol..

[61] Yaffe H., Ron M., Polishuk W. (1978). Amenorrhoea, hypomenorrhoea and uterine fibrosis. Am. J. Obstet. Gynecol..

[62] Han Q., Du Y. (2020). Advances in the application of biomimetic endometrium interfaces for uterine bioengineering in female infertility. Front. Bioeng. Biotechnol..

[63] Wei C., Pan Y., Zhang Y., Dai Y., Jiang L., Shi L., Yang W., Xu S., Zhang Y., Xu W. (2020). Overactivated sonic hedgehog signaling aggravates intrauterine adhesion via inhibiting autophagy in endometrial stromal cells. Cell. Death Dis..

[64] Liu F., Hu S., Wang S., Cheng K. (2019). Cell and biomaterial-based approaches to uterus regeneration. Regen. Biomater..

[65] Maybin J.A., Critchley H. (2015). Menstrual physiology: Implications for endometrial pathology and beyond. Hum. Reprod. Update.

[66] Chen G., Liu L., Sun J., Zeng L., Cai H., He Y. (2020). Foxf2 and Smad6 co-regulation of collagen 5A2 transcription is involved in the pathogenesis of intrauterine adhesion. J. Cell. Mol. Med..

[67] Guo L.P., Chen L.M., Chen F., Jiang N.H., Sui L. (2019). Smad signaling coincides with epithelial-mesenchymal transition in a rat model of intrauterine adhesion. Am. J. Transl. Res..

[68] Zhou Q., Wu X., Hu J., Yuan R. (2018). Abnormal expression of fibrosis markers, estrogen receptor α and stromal derived factor-1/chemokine (C-X-C motif) receptor-4 axis in intrauterine adhesions. Int. J. Mol. Med..

[69] Xu Q., Duan H., Gan L., Liu X., Chen F., Shen X., Tang Y.Q., Wang S. (2017). MicroRNA-1291 promotes endometrial fibrosis by regulating the ArhGAP29-RhoA/ROCK1 signaling pathway in a murine model. Mol. Med. Rep..

[70] Salma U., Xue M., Ali Sheikh M.S., Guan X., Xu B., Zhang A., Huang L., Xu D. (2016). Role of transforming growth factor-β1 and smads signaling pathway in intrauterine adhesion. Mediat. Inflamm..

[71] Liu X., Duan H., Zhang H.H., Gan L., Xu Q. (2016). Integrated data set of microRNAs and mRNAs involved in severe intrauterine adhesion. Reprod. Sci..

[72] Santamaria X., Isaacson K., Simón C. (2018). Asherman’s Syndrome: It may not be all our fault. Hum. Reprod..

[73] Friedman J.A., Wong J.M.K., Chaudhari A., Tsai S., Milad M.P. (2018). Hysteroscopic myomectomy: A comparison of techniques and review of current evidence in the management of abnormal uterine bleeding. Curr. Opin. Obstet. Gynecol..

[74] Healy M.W., Schexnayder B., Connell M.T., Terry N., DeCherney A.H., Csokmay J.M., Yauger B.J., Hill M.J. (2016). Intrauterine adhesion prevention after hysteroscopy: A systematic review and meta-analysis. Am. J. Obstet. Gynecol..

[75] Haber K., Hawkins E., Levie M., Chudnoff S. (2015). Hysteroscopic morcellation: Review of the manufacturer and user facility device experience (MAUDE) database. J. Minim. Invasive Gynecol..

[76] Ciebiera M., Łoziński T., Wojtyła C., Rawski W., Jakiel G. (2018). Complications in modern hysteroscopic myomectomy. Ginekol. Polska.

[77] Capmas P., Levaillant J.M., Fernandez H. (2013). Surgical techniques and outcome in the management of submucous fibroids. Curr. Opin. Obstet. Gynecol..

[78] Gambadauro P., Gudmundsson J., Torrejón R. (2012). Intrauterine adhesions following conservative treatment of uterine fibroids. Obstet. Gynecol. Int..

[79] Litta P., Leggieri C., Conte L., Dalla Toffola A., Multinu F., Angioni S. (2014). Monopolar versus bipolar device: Safety, feasibility, limits and perioperative complications in performing hysteroscopic myomectomy. Clin. Exp. Obstet. Gynecol..

[80] Hamerlynck T.W., van Vliet H.A., Beerens A.S., Weyers S., Schoot B.C. (2016). Hysteroscopic morcellation versus loop resection for removal of placental remnants: A randomized trial. J. Minim. Invasive Gynecol..

[81] Mazzon I., Favilli A., Cocco P., Grasso M., Horvath S., Bini V., Di Renzo G.C., Gerli S. (2014). Does cold loop hysteroscopic myomectomy reduce intrauterine adhesions? A retrospective study. Fertil. Steril..

[82] Van Wessel S., van Vliet H.A.A.M., Schoot B.C., Weyers S., Hamerlynck T.W.O. (2021). Hysteroscopic morcellation versus bipolar resection for removal of type 0 and 1 submucous myomas: A randomized trial. Eur. J. Obstet. Gynecol. Reprod. Biol..

[83] Chua K.J.C., McLucas B. (2021). Sepsis following hysteroscopic myomectomy. Minim. Invasive Ther. Allied Technol..

[84] Capmas P., Lobersztajn A., Duminil L., Barral T., Pourcelot A.G., Fernandez H. (2019). Operative hysteroscopy for retained products of conception: Efficacy and subsequent fertility. J. Gynecol. Obstet. Hum. Reprod..

[85] Barel O., Krakov A., Pansky M., Vaknin Z., Halperin R., Smorgick N. (2015). Intrauterine adhesions after hysteroscopic treatment for retained products of conception: What are the risk factors?. Fertil. Steril..

[86] Huang C.Y., Chang W.H., Cheng M., Huang H.Y., Horng H.C., Chen Y.J., Lee W.L., Wang P.H. (2020). Crosslinked hyaluronic acid gels for the prevention of intrauterine adhesions after a hysteroscopic myomectomy in women with submucosal myomas: A prospective, randomized, controlled trial. Life.

[87] Zhao H., Yang B., Li H., Xu Y., Feng L. (2019). Successful pregnancies in women with diffuse uterine leiomyomatosis after hysteroscopic management using the hysteroscopy endo operative system. J. Minim. Invasive Gynecol..

[88] Vitale S.G., Sapia F., Rapisarda A.M.C., Valenti G., Santangelo F., Rossetti D., Chiofalo B., Sarpietro G., La Rosa V.L., Triolo O. (2017). Hysteroscopic morcellation of submucous myomas: A systematic review. Biomed. Res. Int..

[89] Hsu Y.H., Yeh C.C., Wang P.H. (2019). The better way-uterine feeding vessel occlusion to manage postpartum hemorrhage. Taiwan. J. Obstet. Gynecol..

[90] Chao H.T., Wang P.H. (2014). Fertility outcomes after uterine artery occlusion in the management of women with symptomatic uterine fibroids. Taiwan. J. Obstet. Gynecol..

[91] Lee W.L., Liu W.M., Fuh J.L., Tsai Y.C., Shih C.C., Wang P.H. (2010). Use of uterine vessel occlusion in the management of uterine myomas: Two different approaches. Fertil. Steril..

[92] Song D., Liu Y., Xiao Y., Li T.C., Zhou F., Xia E. (2014). A matched cohort study comparing the outcome of intrauterine adhesiolysis for Asherman’s syndrome after uterine artery embolization or surgical trauma. J. Minim. Invasive Gynecol..

[93] Jiang J., Wang C., Xue M. (2020). High-intensity focused ultrasound versus uterine artery embolization for patients with retained placenta accrete. Eur. J. Obstet. Gynecol. Reprod. Biol..

[94] Orlando M., Kollikonda S., Hackett L., Kho R. (2021). Nonhysteroscopic myomectomy and fertility outcomes: A systematic review. J. Minim. Invasive Gynecol..

[95] Sancho J.M., Delgado V.S.C., Valero M.J.N., Soteras M.G., Amate V.P., Carrascosa A.A. (2016). Hysteroscopic myomectomy outcomes after 3-month treatment with either Ulipristal Acetate or GnRH analogues: A retrospective comparative study. Eur. J. Obstet. Gynecol. Reprod. Biol..

[96] Murji A., Wais M., Lee S., Pham A., Tai M., Liu G. (2018). A multicenter study evaluating the effect of ulipristal acetate during myomectomy. J. Minim. Invasive Gynecol..

[97] Sayyah-Melli M., Bidadi S., Taghavi S., Ouladsahebmadarek E., Jafari-Shobeiri M., Ghojazadeh M., Rahmani V. (2016). Comparative study of vaginal danazol vs. diphereline (a synthetic GnRH agonist) in the control of bleeding during hysteroscopic myomectomy in women with abnormal uterine bleeding: A randomized controlled clinical trial. Eur. J. Obstet. Gynecol. Reprod. Biol..

[98] Cheng M.H., Wang P.H. (2008). Uterine myoma: A condition amenable to medical therapy?. Expert Opin. Emerg. Drugs.

[99] Wang P.H., Lee W.L., Cheng M.H., Yen M.S., Chao K.C., Chao H.T. (2009). Use of a gonadotropin-releasing hormone agonist to manage perimenopausal women with symptomatic uterine myomas. Taiwan. J. Obstet. Gynecol..

[100] Coddington C.C., Grow D.R., Ahmed M.S., Toner J.P., Cook E., Diamond M.P. (2009). Gonadotropin-releasing hormone agonist pretreatment did not decrease postoperative adhesion formation after abdominal myomectomy in a randomized control trial. Fertil. Steril..

[101] Acunzo G., Guida M., Pellicano M., Tommaselli G.A., Di Spiezio Sardo A., Bifulco G., Cirillo D., Taylor A., Nappi C. (2003). Effectiveness of auto-cross-linked hyaluronic acid gel in the prevention of intrauterine adhesions after hysteroscopic adhesiolysis: A prospective, randomized, controlled study. Hum. Reprod..

[102] Zhou Q., Shi X., Saravelos S., Huang X., Zhao Y., Huang R., Xia E., Li T.C. (2021). Auto-cross-linked hyaluronic acid gel for prevention of intrauterine adhesions after hysteroscopic adhesiolysis: A randomized controlled trial. J. Minim. Invasive Gynecol..

[103] Bosteels J., Weyers S., D’Hooghe T.M., Torrance H., Broekmans F.J., Chua S.J., Mol B.W.J. (2017). Anti-Adhesion therapy following operative hysteroscopy for treatment of female subfertility. Cochrane Database Syst. Rev..

[104] Lin X., Wei M., Li T.C., Huang Q., Huang D., Zhou F., Zhang S. (2013). A comparison of intrauterine balloon, intrauterine contraceptive device and hyaluronic acid gel in the prevention of adhesion reformation following hysteroscopic surgery for Asherman syndrome: A cohort study. Eur. J. Obstet. Gynecol. Reprod. Biol..

[105] Salma U., Xue M., Md Sayed A.S., Xu D. (2014). Efficacy of intrauterine device in the treatment of intrauterine adhesions. Biomed. Res. Int..

[106] Azumaguchi A., Henmi H., Saito T. (2019). Efficacy of silicone sheet as a personalized barrier for preventing adhesion reformation after hysteroscopic adhesiolysis of intrauterine adhesions. Reprod. Med. Biol..

[107] Zhu R., Duan H., Gan L., Wang S. (2018). Comparison of intrauterine suitable balloon and Foley balloon in the prevention of adhesion after hysteroscopic adhesiolysis. Biomed. Res. Int..

[108] Huang H., Xu B., Cheng C., Xu D. (2020). A novel intrauterine stent for prevention of intrauterine adhesions. Ann. Transl. Med..

[109] Fuchs N., Smorgick N., Ben Ami I., Vaknin Z., Tovbin Y., Halperin R., Pansky M. (2014). Intercoat (Oxiplex/AP gel) for preventing intrauterine adhesions after operative hysteroscopy for suspected retained products of conception: Double-blind, prospective, randomized pilot study. J. Minim. Invasive Gynecol..

[110] Di Spiezio Sardo A., Spinelli M., Bramante S., Scognamiglio M., Greco E., Guida M., Cela V., Nappi C. (2011). Efficacy of a polyethylene oxide-sodium carboxymethylcellulose gel in prevention of intrauterine adhesions after hysteroscopic surgery. J. Minim. Invasive Gynecol..

[111] Bosteels J., Weyers S., Mol B.W., D’Hooghe T. (2014). Anti-Adhesion barrier gels following operative hysteroscopy for treating female infertility: A systematic review and meta-analysis. Gynecol. Surg..

[112] Bosteels J. (2017). Anti-Adhesion barrier gels: Time for evidence-informed practice in gynecologic surgery?. Fertil. Steril..

[113] Thubert T., Dussaux C., Demoulin G., Rivain A.L., Trichot C., Deffieux X. (2015). Influence of auto-cross-linked hyaluronic acid gel on pregnancy rate and hysteroscopic outcomes following surgical removal of intra-uterine adhesions. Eur. J. Obstet. Gynecol. Reprod. Biol..

[114] Hooker A.B., de Leeuw R., van de Ven P.M., Bakkum E.A., Thurkow A.L., Vogel N.E.A., van Vliet H.A.A.M., Bongers M.Y., Emanuel M.H., Verdonkschot A.E.M. (2017). Prevalence of intrauterine adhesions after the application of hyaluronic acid gel after dilatation and curettage in women with at least one previous curettage: Short-term outcomes of a multicenter, prospective randomized controlled trial. Fertil. Steril..

[115] Hooker A.B., de Leeuw R.A., van de Ven P.M., Brölmann H.A.M., Huirne J.A.F. (2018). Reproductive performance after the application of hyaluronic acid gel after dilation and curettage in women who have experienced at least one previous curettage: Long-term results of a multicenter prospective randomized trial. Fertil. Steril..

[116] Hooker A.B., de Leeuw R.A., van de Ven P.M., Brölmann H.A.M., Huirne J.A. (2020). Pregnancy and neonatal outcomes 42 months after application of hyaluronic acid gel following dilation and curettage for miscarriage in women who have experienced at least one previous curettage: Follow-up of a randomized controlled trial. Fertil. Steril..

[117] Can S., Kirpinar G., Dural O., Karamustafaoglu B.B., Tas I.S., Yasa C., Ugurlucan F.G. (2018). Efficacy of a new crosslinked hyaluronan gel in the prevention of intrauterine adhesions. J.S.L.S..

[118] Mais V., Cirronis M.G., Peiretti M., Ferrucci G., Cossu E., Melis G.B. (2012). Efficacy of auto-crosslinked hyaluronan gel for adhesion prevention in laparoscopy and hysteroscopy: A systematic review and meta-analysis of randomized controlled trials. Eur. J. Obstet. Gynecol. Reprod. Biol..

[119] Guida M., Acunzo G., Di Spiezio Sardo A., Bifulco G., Piccoli R., Pellicano M., Cerrota G., Cirillo D., Nappi C. (2004). Effectiveness of auto-crosslinked hyaluronic acid gel in the prevention of intrauterine adhesions after hysteroscopic surgery: A prospective, randomized, controlled study. Hum. Reprod..

[120] Kim T., Ahn K.H., Choi D.S., Hwang K.J., Lee B.I., Jung M.H., Kim J.W., Kim J.H., Cha S.H., Lee K.H. (2012). A randomized, multi-center, clinical trial to assess the efficacy and safety of alginate carboxymethylcellulose hyaluronic acid compared to carboxymethylcellulose hyaluronic acid to prevent postoperative intrauterine adhesion. J. Minim. Invasive Gynecol..

[121] Hindocha A., Beere L., Dias S., Watson A., Ahmad G. (2015). Adhesion prevention agents for gynaecological surgery: An overview of Cochrane reviews. Cochrane Database Syst. Rev..

[122] Zheng F., Zhu B., Xin X., He F., Cui Y. (2019). Meta-Analysis on the use of hyaluronic acid gel to prevent recurrence of intrauterine adhesion after hysteroscopic adhesiolysis. Taiwan. J. Obstet. Gynecol..

[123] Zheng F., Xin X., He F., Cui Y. (2020). Meta-Analysis of the use of hyaluronic acid gel to prevent intrauterine adhesions after miscarriage. Eur. J. Obstet. Gynecol. Reprod. Biol..

[124] Johary J., Xue M., Zhu X., Xu D., Velu P.P. (2014). Efficacy of estrogen therapy in patients with intrauterine adhesions: Systematic review. J. Minim. Invasive Gynecol..

[125] Zheng F., Xin X., He F., Liu J., Cui Y. (2020). Meta-Analysis on the use of hyaluronic acid gel to prevent intrauterine adhesion after intrauterine operations. Exp. Ther. Med..

[126] Shokeir T.A., Fawzy M., Tatongy M. (2008). The nature of intrauterine adhesions following reproductive hysteroscopic surgery as determined by early and late follow-up hysteroscopy: Clinical implications. Arch. Gynecol. Obstet..

[127] Sebbag L., Even M., Fay S., Naoura I., Revaux A., Carbonnel M., Pirtea P., de Ziegler D., Ayoubi J.M. (2019). Early second-look hysteroscopy: Prevention and treatment of intrauterine post-surgical adhesions. Front. Surg..

[128] Li X., Wu L., Zhou Y., Fan X., Huang J., Wu J., Yu R., Lou J., Yang M., Yao Z. (2019). New crosslinked hyaluronan gel for the prevention of intrauterine adhesions after dilation and curettage in patients with delayed miscarriage: A prospective, multicenter, randomized, controlled trial. J. Minim. Invasive Gynecol..

[129] Tsapanos V.S., Stathopoulou L.P., Papathanassopoulou V.S., Tzingounis V.A. (2002). The role of Seprafilm bioresorbable membrane in the prevention and therapy of endometrial synechiae. J. Biomed. Mater. Res..

[130] Xiao S., Wan Y., Zou F., Ye M., Deng H., Ma J., Wei Y., Tan C., Xue M. (2015). Prevention of intrauterine adhesion with auto-crosslinked hyaluronic acid gel: A prospective, randomized, controlled clinical study. Zhonghua. Fu Chan Ke Za Zhi.

[131] Ducarme G., Davitian C., Zarrouk S., Uzan M., Poncelet C. (2006). Interest of auto-crosslinked hyaluronic acid gel in the prevention of intrauterine adhesions after hysteroscopic surgery: A case-control study. J. Gynecol. Obstet. Biol. Reprod..

[132] Krajcovicova R., Hudeck R., Ventruba P., Surgentova K. (2015). The role of hyaluronan in Asherman’s syndrome therapy. J. Gynecol. Surg..

[133] Salama N.M., Zaghlol S.S., Mohamed H.H., Kamar S.S. (2020). Suppression of the inflammation and fibrosis in asherman syndrome rat model by mesenchymal stem cells: Histological and immunohistochemical studies. Folia Histochem. Cytobiol..

[134] Cao J., Liu D., Zhao S., Yuan L., Huang Y., Ma J., Yang Z., Shi B., Wang L., Wei J. (2020). Estrogen attenuates TGF-β1-induced EMT in intrauterine adhesion by activating Wnt/β-catenin signaling pathway. Braz. J. Med. Biol. Res..

[135] Matsubara S. (2020). A novel uterine stent for preventing intrauterine adhesion: Not only gynecologic but also obstetric significance. Ann. Transl. Med..

[136] Xin L., Lin X., Zhou F., Li C., Wang X., Yu H., Pan Y., Fei H., Ma L., Zhang S. (2020). A scaffold laden with mesenchymal stem cell-derived exosomes for promoting endometrium regeneration and fertility restoration through macrophage immunomodulation. Acta Biomater..

[137] Ai Y., Chen M., Liu J., Ren L., Yan X., Feng Y. (2020). lncRNA TUG1 promotes endometrial fibrosis and inflammation by sponging miR-590-5p to regulate Fasl in intrauterine adhesions. Int. Immunopharmacol..

[138] Zhang Z., Li S., Deng J., Yang S., Xiang Z., Guo H., Xi H., Sang M., Zhang W. (2020). Aspirin inhibits endometrial fibrosis by suppressing the TGF-β1-Smad2/Smad3 pathway in intrauterine adhesions. Int. J. Mol. Med..

[139] De Miguel-Gómez L., López-Martínez S., Campo H., Francés-Herrero E., Faus A., Díaz A., Pellicer A., Domínguez F., Cervelló I. (2021). Comparison of different sources of platelet-rich plasma as treatment option for infertility-causing endometrial pathologies. Fertil. Steril..

[140] Queckbörner S., Davies L.C., von Grothusen C., Santamaria X., Simón C., Gemzell-Danielsson K. (2019). Cellular therapies for the endometrium: An update. Acta. Obstet. Gynecol. Scand..

[141] Park M., Hong S.H., Park S.H., Kim Y.S., Yang S.C., Kim H.R., Noh S., Na S., Lee H.K., Lim H.J. (2020). Perivascular stem cell-derived cyclophilin A improves uterine environment with Asherman’s syndrome via HIF1α-dependent angiogenesis. Mol. Ther..

[142] Bozorgmehr M., Gurung S., Darzi S., Nikoo S., Kazemnejad S., Zarnani A.H., Gargett C.E. (2020). Endometrial and menstrual blood mesenchymal stem/stromal cells: Biological properties and clinical application. Front. Cell. Dev. Biol..

[143] Gan L., Duan H., Sun F.Q., Xu Q., Tang Y.Q., Wang S. (2017). Efficacy of freeze-dried amnion graft following hysteroscopic adhesiolysis of severe intrauterine adhesions. Int. J. Gynaecol. Obstet..

[144] Lee D.Y., Lee S.R., Kim S.K., Joo J.K., Lee W.S., Shin J.H., Cho S., Park J.C., Kim S.H. (2020). A new thermo-responsive hyaluronic acid sol-gel to prevent intrauterine adhesions after hysteroscopic surgery: A randomized, non-inferiority trial. Yonsei Med. J..

[145] Tonguc E.A., Var T., Yilmaz N., Batioglu S. (2010). Intrauterine device or estrogen treatment after hysteroscopic uterine septum resection. Int. J. Gynaecol. Obstet..

[146] Yu X., Yuhan L., Dongmei S., Enlan X., Tinchiu L. (2016). The incidence of post-operative adhesion following transection of uterine septum: A cohort study comparing three different adjuvant therapies. Eur. J. Obstet. Gynecol. Reprod. Biol..

[147] Vercellini P., Fedele L., Arcaini L., Rognoni M.T., Candiani G.B. (1989). Value of intrauterine device insertion and estrogen administration after hysteroscopic metroplasty. J. Reprod. Med..

[148] Cheng M., Chang W.H., Yang S.T., Huang H.Y., Tsui K.H., Chang C.P., Lee W.L., Wang P.H. (2020). Efficacy of applying hyaluronic acid gels in the primary prevention of intrauterine adhesion after hysteroscopic myomectomy: A meta-analysis of randomized controlled trials. Life.

[149] De Iaco P.A., Muzzupapa G., Bovicelli A., Marconi S., Bitti S.R., Sansovini M., Bovicelli L. (2003). Hyaluronan derivative gel in intrauterine adhesion (IUA) prevention after operative hysteroscopy. Ellipse.

[150] Şükür Y.E., Saridogan E. (2021). Multiple myomectomy to aid fertility treatment—Surgical and fertility outcomes: A retrospective cohort study. Facts Views Vis. ObGyn.

[151] Li C., Wang W., Sun S., Xu Y., Fang Z., Cong L. (2021). Expression and potential role of MMP-9 in intrauterine adhesion. Mediat. Inflamm..

[152] Zhao X., Gao B., Yang X., Zhang A., Jamail G., Li Y., Xu D. (2021). The density of endometrial glandular openings: A novel variable to predict the live birth rate in patients with intrauterine adhesions following hysteroscopic adhesiolysis. Hum. Reprod..

[153] Zhang M., Lin X. (2021). Analysis of risk factors for obstetric outcomes after hysteroscopic adhesiolysis for Asherman syndrome: A retrospective cohort study. Int. J. Gynaecol. Obstet..

[154] Zhang T., Zhang R., Li J., Tang J., Shen C., Shen L., Dong J., Zhang X. (2021). Preparation of fibroblast suppressive poly(ethylene glycol)-*b*-poly(l-phenylalanine)/poly(ethylene glycol) hydrogel and its application in intrauterine fibrosis prevention. ACS Biomater. Sci. Eng..

[155] Abbott J., Deans R. (2021). Accelerating the science after 125 years of treating intrauterine adhesions. J. Minim. Invasive Gynecol..

[156] Hooker A.B., de Leeuw R.A., Twisk J.W.R., Brölmann H.A.M., Huirne J.A.F. (2021). Reproductive performance of women with and without intrauterine adhesions following recurrent dilatation and curettage for miscarriage: Long-term follow-up of a randomized controlled trial. Hum. Reprod..

[157] Lee W.L., Lee F.K., Wang P.H. (2021). Application of hyaluronic acid in patients with interstitial cystitis. J. Chin. Med. Assoc..

[158] Tsai C.P., Yang J.M., Liang S.J., Lin Y.H., Huang W.C., Lin T.Y., Hsu C.S., Chuang F.C., Hung M.J. (2021). Factors associated with treatment outcomes after intravesical hyaluronic acid therapy in women with refractor interstitial cystitis: A prospective, multicenter study. J. Chin. Med. Assoc..

[159] Peng Y.C., Yueh-Hsia Chiu S., Feng M., Liang C.C. (2020). The effect of intravesical hyaluronic acid therapy on urodynamic and clinical outcomes among women with interstitial cystitis/bladder pain syndrome. Taiwan. J. Obstet. Gynecol..

[160] Gote V., Sharma A.D., Pal D. (2021). Hyaluronic acid-targeted stimuli-sensitive nanomicelles co-encapsulating paclitaxel and ritonavir to overcome multi-drug resistance in metastatic breast cancer and triple-negative breast cancer cells. Int. J. Mol. Sci..

[161] Chang M.C., Chiang P.F., Kuo Y.J., Peng C.L., Chen K.Y., Chiang Y.C. (2021). Hyaluronan-Loaded liposomal dexamethasone-diclofenac nanoparticles for local osteoarthritis treatment. Int. J. Mol. Sci..

[162] Kim S.B., Cho J., Jue S.S., Park J.H., Kim J.Y. (2020). Effect of hyaluronic acid filler injection on the interdental papilla in a mouse model of open gingival embrasure. Int. J. Environ. Res. Public Health.

[163] Winter C., Keimel R., Gugatschka M., Kolb D., Leitinger G., Roblegg E. (2021). Investigation of changes in saliva in radiotherapy-induced head neck cancer patients. Int. J. Environ. Res. Public Health.

[164] Benor A., Gay S., DeCherney A. (2020). An update on stem cell therapy for Asherman syndrome. J. Assist. Reprod. Genet..

[165] Jiang X., Li X., Fei X., Shen J., Chen J., Guo M., Li Y. (2021). Endometrial membrane organoids from human embryonic stem cell combined with the 3D Matrigel for endometrium regeneration in asherman syndrome. Bioact. Mater..

[166] Lee S.Y., Shin J.E., Kwon H., Choi D.H., Kim J.H. (2020). Effect of autologous adipose-derived stromal vascular fraction transplantation on endometrial regeneration in patients of Asherman’s syndrome: A pilot study. Reprod. Sci..

[167] Zhang S., Chang Q., Li P., Tong X., Feng Y., Hao X., Zhang X., Yuan Z., Tan J. (2021). Concentrated small extracellular vesicles from menstrual blood-derived stromal cells improve intrauterine adhesion, a pre-clinical study in a rat model. Nanoscale.

[168] Zhao Y.X., Chen S.R., Huang Q.Y., Chen W.C., Xia T., Shi Y.C., Gao H.Z., Shi Q.Y., Lin S. (2021). Repair abilities of mouse autologous adipose-derived stem cells and ShakeGel™3D complex local injection with intrauterine adhesion by BMP7-Smad5 signaling pathway activation. Stem Cell Res. Ther..

